# Development of an Index for the Inspection of Aedes aegypti Breeding Sites in Brazil: Multi-criteria Analysis

**DOI:** 10.2196/19502

**Published:** 2021-05-10

**Authors:** Yuri Lima, Wallace Pinheiro, Carlos Eduardo Barbosa, Matheus Magalhães, Miriam Chaves, Jano Moreira de Souza, Sérgio Rodrigues, Geraldo Xexéo

**Affiliations:** 1 Graduate School of Engineering (COPPE) Federal University of Rio de Janeiro (UFRJ) Rio de Janeiro Brazil; 2 Military School of Engineering (IME) Rio de Janeiro Brazil; 3 Center of Analysis of Naval Systems (CASNAV) Brazilian Navy Rio de Janeiro Brazil; 4 National Laboratory of Scientific Computation Brazilian Ministry of Science, Technology, Innovation, and Communications Petrópolis Brazil; 5 Lemobs Rio de Janeiro Brazil

**Keywords:** multi-criteria analysis, public health, human sensors, vector surveillance, tropical diseases

## Abstract

**Background:**

*Aedes aegypti* is a vector for the transmission of diseases such as dengue fever, chikungunya, Zika fever, and yellow fever. In 2016, over 1 million cases of these diseases were reported in Brazil, which is an alarming public health issue. One of the ways of controlling this disease is by inspecting and neutralizing the places where *A. aegypti* lays its eggs. The Ministry of Planning, Development, and Administration of Brazil maintains the inspection statistics.

**Objective:**

We propose a multi-criteria analysis to create an index for *A. aegypti* inspections reported through the Ministry of Planning, Development, and Administration system of Brazil.

**Methods:**

Based on the repository from urban cleaning services combined with data on inspections conducted by government agencies in several Brazilian cities and municipalities, we selected and combined metrics, which we further ranked using the analytic hierarchy process methodology. We also developed risk maps based on the analytic hierarchy process ranking of the *A. aegypti* breeding sites.

**Results:**

Based on our analysis and the available data, the priority for inspections should consider the number of sick people (weight 0.350), medical evaluations (weight 0.239), inspections (weight 0.201), mosquito breeding sites (weight 0.126), and days of absence from work (weight 0.096).

**Conclusions:**

The proposed index could aid public health practitioners in preventing the appearance of new *A. aegypti* breeding sites. This information technology application can help solve such public health challenges.

## Introduction

### Background

*Aedes aegypti* is a vector for many diseases such as chikungunya, dengue fever, yellow fever, and Zika virus. The control of these diseases is difficult as there are several hard-to-find mosquito breeding places (eg, empty bottles, plant vases, car tires) that can be found at any abandoned lot or in any house. In Brazil, *A. aegypti* is the main vector for dengue fever. However, *Aedes albopictus* is also a vector of such arboviruses [1] and have been reported in Brazil since 1986 [2]. *A. aegypti* and *A. albopictus* tend to breed in similar sites, and researchers have documented competition between these species. *A. aegypti* is more prevalent in densely populated highly urbanized areas, while *A. albopictus* is more prevalent in rural, suburban, and forested urban areas [2].

Although *A. albopictus* is still regarded as the potential vector of arboviruses in most countries of the Americas [3], our work focused mostly in urban areas, and the objective of this study is the control of the breeding of *A. aegypti*. Nevertheless, our work can be applied with minor modifications to the control of breeding of *A. albopictus*. The challenge in controlling this vector is one of the reasons for the significant numbers associated with the outbreaks of the diseases, which shows how important it is as a public health issue. There are approximately 390 million cases estimated each year for dengue alone [4]. In Brazil, in 2016, there were over 1 million cases of diseases caused by *A. aegypti* [5].

There are currently several methods to control the breeding of *A. aegypti*, which has led to a reduction in the cases of disease related to it. These methods are usually combined and include contaminating the female mosquitoes with *Wolbachia* strains so that they cannot spread diseases [6,7], eliminating mosquitoes using poison, controlling breeding sites through inspections and simple measures (eg, putting sand in vases, covering water recipients, storing empty bottles upside down), and neutralizing the breeding sites found.

In Brazil, the *A. aegypti* infestation index rapid survey (*Levantamento Rápido de Índices para Aedes aegypti*) is a simplified entomological surveillance method adopted by the Ministry of Health to determine the infestation rates of *A. aegypti*. Municipalities perform larval surveys through systematic sampling of buildings to calculate the Breteau index and the building infestation index. The sample depends on the population density and the number of buildings. The indexes predominantly identify breeding sites and are used as indicators to initiate actions to neutralize these sites and reduce the use of larvicides [8].

Controlling *A. aegypti* is a transdisciplinary effort, and information technology plays a role, mainly in the creation of a data repository to support analysis and decision making. An example of information technology is the Sigelu Aedes software developed by the Brazilian company Lemobs as commissioned by the Brazilian Ministry of Planning, Development, and Administration. This software helps public workers to inspect government buildings reporting *Aedes* breeding sites and the actions taken to control them.

### Goal of This Study

Given this context, the goal of our work is to contribute to the effort of controlling *A. aegypti* breeding sites by proposing a georeferenced index that helps decision makers to identify places where mosquitoes could breed. We used a multi-criteria analysis, more specifically the analytic hierarchy process (AHP), to develop such an index. We tested our index by using real data from the Ministry of Planning, Development, and Administration of Brazil to produce a risk map layer in a multidimensional data model.

### Multi-criteria Analysis in Disease Vector Surveillance

Multi-criteria analysis has been used previously in disease vector surveillance literature for supporting decision making. We review such related literature with a special focus on the methodology and outcomes of each study. Aenishaenslin et al [9] identified, evaluated, and ranked different strategies for Lyme disease management in Quebec. They identified 3 intervention areas (preventive communication strategies, surveillance strategies, and control strategies) but focused on the latter two. They defined a multi-criteria decision analysis (MCDA) process with 10 steps—the first 7 focus on problem structuring and the last 3 on the decision analysis. Stakeholders were involved in the identification of issues, definition of criteria, selection of interventions, and individual weighting of the criteria defined. The authors used DSight software to perform the Preference Ranking Organization Method for Enrichment of Evaluations (PROMETHEE) and produce a visual model (geometrical analysis for interactive aid [GAIA]) to display the analysis results. Finally, they produced group rankings analysis and assessed the performance of the selected interventions.

In a second study, Aenishaenslin et al [10] adapted and evaluated the decision model, which had been constructed to rank interventions for Lyme disease prevention in Quebec [9] for a different epidemiological context in Switzerland, where Lyme disease has been endemic for over 30 years. They used a group of Swiss stakeholders to define a new set of criteria to evaluate the problem. The stakeholders kept the original criteria from Quebec but added 4 new criteria. They subsequently performed the analysis by comparing the resulting criteria sets; however, the original criteria had their weights normalized so that their relative importance was in line with the Swiss stakeholders.

Cox et al [11] designed a standardized method to prioritize infectious diseases of humans and animals that may emerge due to the climate change in Canada. They developed user-friendly tools to aid pathogen prioritization, which provided a structure for the study and enabled the decision-making process to be recorded. They identified 40 criteria (from the published literature and experts) that might be used to prioritize potential emerging pathogens in Canada and divided them into 5 groups—3 criterion groups measure the likelihood of pathogen emergence in Canada and 2 criterion groups measure the pathogen impact. Expert opinion was used in the criterion selection and weighting. The weights of all the criteria were standardized. Cox et al [11] then used 2 tools: a spreadsheet developed in Microsoft Excel and the MACBETH tool present in the M-MACBETH software [12]. Santos et al [13] developed a knowledge-driven spatial model to identify risk areas for foot-and-mouth disease (FMD) occurrence—this disease affects cloven-hoofed livestock and wildlife. They evaluated the FMD surveillance performance in the southern Brazilian state of Rio Grande do Sul by using MCDA. Thirteen experts analyzed 18 variables associated with FMD introduction and dissemination pathways. For each pathway, experts defined several risk factors—variables associated with FMD introduction and dissemination. In the next step, the authors requested the experts to weigh each risk factor and pathway following the AHP methodology. The introduction risk and the dissemination risk were calculated separately, and they were then combined multiplicatively, thereby providing the likelihood of FMD occurrence. Finally, the authors built the spatial model using Idrisi 17.0 Selva GIS and Image Processing Software, with raster layers of 1 km × 1 km resolution.

Fruean and East [14] assessed Australia’s targeted surveillance for detecting incursions of the screw-worm fly. Screw-worm fly abundance and survival are affected by—among various criteria—the vegetation type and moisture levels. The authors made a grid of the territory in a map, and to each square, they assigned numeric values for each criterion. They used the multi-criteria analysis shell for spatial decision support package [15], which includes raster maps for feral animal distribution, land use, land cover, and climate data. The authors invited 20 experts to answer a questionnaire in order to assess the relative importance of the potential pathways for the introduction of the screw-worm fly into Australia. Finally, they produced maps of the relative likelihood for the introduction and establishment of the screw-worm fly and considered the seasonal effect of climate. Gosselin et al [16] discussed the implementation of the Integrated System for Public Health Monitoring of West Nile Virus, a real-time geographic information system for public health surveillance of the West Nile virus, in Quebec, Canada. This system gathers information on Corvidae, mosquitoes, humans, horses, climate, and larvicide interventions. It was designed to support the collection, localization, management, and analysis of monitoring data, and presents the results of analyses on maps, tables, and statistical diagrams. Ho et al [17] used a raster-based model to map heat health risks, and they compared this to the traditional vector-based model. Among their goals was the use of the proposed framework to predict and map the heat risk hotspots at multiple spatial scales for the Vancouver area. Heat exposure was estimated using land surface temperature. They used MCDA with 2 different data resampling approaches to visualize the influence of the modifiable areal unit problem (MAUP) issue on heat health risk maps. MAUP is a common source of bias in the results of statistical hypothesis tests in the aggregation of point-based measures. Ho et al [17] used 2 groups of layers: vulnerability layers and heat exposure layers. All vulnerability layers were associated with values ranging from 1 (lowest) to 9 (highest). Each group of layers was combined into a composite layer by assigning equal weights to each layer and normalizing the result. Finally, the composite heat exposure layer was combined with the composite vulnerability layer using the same process. After the modeling, they used the Getis-Ord Gi index [18] to mitigate the problems caused by the MAUP problem.

Hongoh et al [19] ranked possible risk reduction measures for the management of the West Nile virus in Quebec. This study used the methodology presented by Aenishaenslin et al [9]; however, they produced 6 scenarios of increasing potential risk, from low risk (current state) to high risk (epidemics). Hongoh et al [19] elaborated a preliminary list of 15 evaluation criteria in 5 categories. Hongoh et al [19] also used the D-Sight software, the PROMETHEE method, and GAIA maps, and they analyzed interventions at the individual and regional level. Finally, sensitivity analyses were performed on all criteria and for all stakeholders. Sarkar et al [20] performed a five-stage risk assessment for Chagas disease in Texas. First, using Maxent software, they built distribution models for the triatomine species. The environmental layers used were composed of 4 topographical variables and 15 bioclimatic variables. The output of this step was the likelihood of the presence of triatomine. They then did a risk assessment, defining sets of ecological risks and incidence-based risks, which were analyzed using multi-criteria analysis to generate a composite risk. Finally, they combined the composite risk and the population that would be exposed to Chagas disease to produce a relative expected exposure rate.

Thanapongtharm et al [21] characterized the spatial habitat of the flying fox bat, which is a vector of the Nipah virus, in populations along Thailand’s central plain and the mapping zones for potential contact between flying fox bat habitats, pig farms, and human settlements. They collected geographic information about flying fox colonies—generally located in areas such as Buddhist temples, surrounded by bodies of water and vegetation—and combined them with the following layers: water bodies, human population density, elevation, and land cover. Thanapongtharm et al [21] used 7 species distribution models to map the ecological suitability for flying fox colonies. Their models were subject to 10 bootstraps to prevent overfitting and due to their very low proportion of positive samples in the data set. Finally, they applied potential surface analysis to map the risk area for the Nipah virus, assuming 2 potential scenarios for human infections: humans directly infected from the bats and humans infected through an intermediate pig host.

Vinhaes et al [22] analyzed data on the occurrence of domiciled triatomines—vectors of Chagas disease—in non-Amazonian regions of Brazil. MCDA was used to assess municipalities’ vulnerability based on socioeconomic, demographic, entomological, and environmental indicators. The program to support decision making based on indicators (PRADIN) software [23], which implements the PROMETHEE II algorithm, was used for MCDA. The authors conducted 6 simulations using PRADIN, in which the municipalities were ranked and classified into quintiles, and their geographic coordinates were then used in the TerraView [24] software to produce vulnerability maps for the occurrence of Chagas disease transmission via domiciled triatomines that were compared to acute vector-borne Chagas disease cases between 2001 and 2012.

## Methods

Our proposed approach involves performing an analysis to create an index of the inspections to find places where *A. aegypti* lays their eggs. The data source for this study is the Ministry of Planning, Development, and Administration of Brazil through the Sigelu Aedes software. Like Santos et al [13], we adopted MCDA because such methods are widely used in the literature. Among MCDA methodologies, we chose to specifically use the AHP methodology. Similar to many other related works [3,6-9,11-14], we produced risk maps, which are useful for disease surveillance and prevention. The proposed approach does not use environmental variables that affect the density and distribution of the mosquito, such as temperature, precipitation, air humidity, and availability of water tanks. Instead, we used indirect indicators, that is, number of inspections, number of sick people, number of medical evaluations, number of days of sickness absence, and number of mosquito breeding sites found. Although the use of nontraditional data sources to trace arbovirus transmission can be found in the literature [25], our approach provides a novel paradigm, since these governmental data can provide an independent indicator, which may be applied in conjunction with the current methodologies. The proposed approach also takes into account that mosquitoes can repopulate sites that have been inspected. We also consider that information about inspections may indicate regions where it is more likely to find mosquitoes. A new index based on this information may help to plan actions to fight against this vector of many diseases in cities. We created the new index using some metrics extracted from the repository.

Managers of urban cleaning services provide data to form a repository. Given that the main breeding sites of *A. aegypti* are abandoned sites that can be used as garbage dumps, the developers of this software decided to integrate resources to combat this mosquito species. Thus, this system contains data on inspections conducted by government agencies in several Brazilian cities and municipalities. However, the metrics used do not indicate possible future mosquito breeding sites —they only record the work done by government agents in the field.

**Figure 1:**
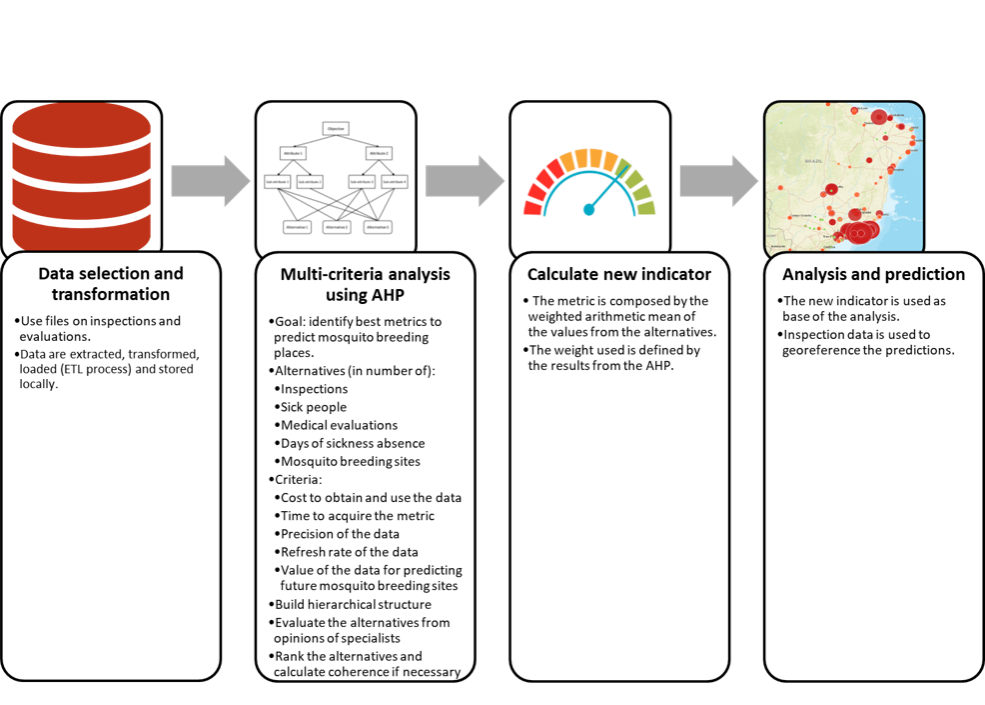
Proposed approach to assist in the prediction of *Aedes aegypti* breeding sites.

Our proposed solution is presented in Figure 1. We compared each of the following alternatives: number of inspections, number of sick people, number of medical evaluations, number of days of sickness absence, and number of mosquito breeding sites. In our comparison, we selected the following criteria to use in the AHP: cost to obtain and use the data, time to acquire the metric, precision of the data, refresh rate of the data, and value of the data for predicting future mosquito breeding sites. For gathering the opinions of specialists about criteria and metrics (alternatives), in accordance with the AHP method, we used the AHP Excel template, with multiple inputs from Goepel [26]. To calculate the matrix for the criteria metrics and matrices for alternatives, we adapted a solution from Griffith [27]. The other calculations are shown in this paper. All inconsistencies related to coherence were less than 0.10, in accordance with the level suggested by Saaty [28]. After determining the final priorities, we assembled the new index, considering the data model that was populated using the repository presented in Figure 1. This set of metrics is used to calculate the new index of an inspection. It is crucial to note that only the metrics related to inspections were used as georeferenced metrics.

## Results

The AHP criteria matrix is shown in Table 1. The prioritization of the criteria is shown in the last column (AHP values are shown in parentheses). The most important criterion was “value,” while the least important was “cost.” The priority value of the “value” criterion was almost double that of the next most important criterion (precision), which indicates the importance of analyzing each metric according to its value. The inconsistency was 0.093.

The matrix for the alternatives with respect to each criterion is presented in Table 2. In the “cost” section, lower values correspond to higher costs. The last column of “priority” shows the ranking of the alternatives. “Number of inspections” was considered the least important, while the “number of sick people” was the most important alternative for costs. In the “time” section, lower values correspond to a longer time. The least important criterion was “number of sick people” and the most important was “number of inspections” (we note that the least and most important criteria are inverted when compared to section “cost”). In the “precision” section, lower values correspond to lower precision. “Number of inspections” occupied the first position again, “number of medical evaluations” was second, and “sick people” was third. In the “value” section, lower values correspond to lower value. The “value” section is the most relevant, because the “value” criterion has higher priority according to Table 2. “Number of sick people” was the most important alternative followed by the “number of medical evaluations.” Finally, in the “refresh rate” section, lower values correspond to lower refresh rates. The alternative “number of days absent” is the most relevant metric.

At this point, it is possible to calculate the final priorities of the alternatives. Firstly, we must multiply the criterion values by the values for the alternatives, as shown in Table 3.

We then obtained the final priorities, which are shown in Table 4. We ended up calculating the weighted arithmetic mean by considering the priorities of the alternatives.

**Table 1:** The analytic hierarchy process criteria matrix.^a^

Criteria	Cost	Time	Precision	Value	Refresh rate	Priority (AHP^b^)
Cost	1	1	0.16	0.11	0.27	5 (0.042)
Time	1	1	0.24	0.17	0.52	4 (0.056)
Precision	1/0.16^c^	1/0.24	1	0.26	7	2 (0.292)
Value	1/0.11	1/0.17	1/0.26	1	4.72	1 (0.516)
Refresh rate	1/0.27	1/0.52	1/7	1/4.72	1	3 (0.094)

^a^The values in this table are the results of a pairwise comparison between each one of the criteria to determine the relative priority of each one.

^b^AHP: analytic hierarchy process value.

^c^The fractions in this table are the result of the pairwise comparisons, which generates a triangular matrix.

**Table 2:** Matrix for alternatives with respect to cost, time, precision, value, and refresh rate.^a^

Alternatives for		Inspections	Sick people	Medical evaluations	Days of absence	Mosquito breeding	Priority (AHP^b^)	Inconsistency^c^	
**Cost**									0.055
	Inspections	1	1/7^d^	1/7	1/7	1/5	5 (0.034)		
	Sick people	1	1	3	3	5	1 (0.459)		
	Medical evaluations	7	1/3	1	1	3	2 (0.207)		
	Days of absence	7	1/3	1	1	3	3 (0.207)		
	Mosquito breeding	5	1/5	1/3	1/3	1	4 (0.094)		
**Time**									0.090
	Inspections	1	7	3	5	3	1 (0.472)		
	Sick people	1/7	1	1/3	1/3	1/3	5 (0.051)		
	Medical evaluations	1/3	3	1	1	3	2 (0.235)		
	Days of absence	1/5	3	1/3	1	3	3 (0.142)		
	Mosquito breeding	1/3	3	1/3	1/3	1	4 (0.099)		
**Precision**									0.095
	Inspections	1	5	3	3	5	1 (0.454)		
	Sick people	1/5	1	1/5	3	1	3 (0.102)		
	Medical evaluations	1/3	5	1	5	3	2 (0.288)		
	Days of absence	1/3	1/3	1/5	1	1	5 (0.074)		
	Mosquito breeding	1/5	1	1/3	1	1	4 (0.082)		
**Value**									0.084
	Inspections	1	1/9	1/7	1/5	1/7	5 (0.029)		
	Sick people	9	1	3	5	5	1 (0.494)		
	Medical evaluations	7	1/3	1	3	3	2 (0.244)		
	Days of absence	5	1/5	1/3	1	1/3	4 (0.086)		
	Mosquito breeding	7	1/5	1/3	3	1	3 (0.147)		
**Refresh rate**									0.055
	Inspections	1	3	1/7	3	1/7	5 (0.042)		
	Sick people	1/3	1	1/3	1/3	1/3	4 (0.056)		
	Medical evaluations	1	3	1	3	3	2 (0.292)		
	Days of absence	1/3	3	1/3	1	1	1 (0.516)		
	Mosquito breeding	1	3	1/3	1	1	3 (0.094)		

^a^The values in this table are the results of a pairwise comparison between each one of the criteria to determine the relative priority of each one.

^b^AHP: analytic hierarchy process value.

^c^The inconsistency value refers to how consistent the opinion of the AHP participants is. If the value is lower than 0.1 or 10%, the inconsistency is acceptable.

^d^The fractions in this table are the results of the pairwise comparisons, which generate a triangular matrix.

**Table 3:** Candidates considering the criteria.^a^

Criterion (A), alternative (B)		A × B	Total	
**Cost (0.042)**			0.042	
	Inspections (0.034)	0.042 × 0.034 = 0.001		
	Sick people (0.459)	0.042 × 0.459 = 0.019		
	Medical evaluations (0.207)	0.042 × 0.207 = 0.009		
	Days of absence (0.207)	0.042 × 0.207 = 0.009		
	Mosquito breeding (0.094)	0.042 × 0.094 = 0.004		
**Time (0.056)**				0.056
	Inspections (0.472)	0.056 × 0.472 = 0.026		
	Sick people (0.051)	0.056 × 0.051 = 0.003		
	Medical evaluations (0.235)	0.056 × 0.235 = 0.013		
	Days of absence (0.142)	0.056 × 0.142 = 0.008		
	Mosquito breeding (0.099)	0.056 × 0.099 = 0.006		
**Precision (0.292)**				0.293
	Inspections (0.454)	0.292 × 0.454 = 0.133		
	Sick people (0.102)	0.292 × 0.102 = 0.030		
	Medical evaluations (0.288)	0.292 × 0.288 = 0.084		
	Days of absence (0.074)	0.292 × 0.074 = 0.022		
	Mosquito breeding (0.082)	0.292 × 0.082 = 0.024		
**Value (0.516)**				0.516
	Inspections (0.029)	0.516 × 0.029 = 0.015		
	Sick people (0.494)	0.516 × 0.494 = 0.255		
	Medical evaluations (0.244)	0.516 × 0.244 = 0.126		
	Days of absence (0.086)	0.516 × 0.086 = 0.044		
	Mosquito breeding (0.147)	0.516 × 0.147 = 0.076		
**Refresh rate (0.094)**				0.105
	Inspections (0.272)	0.094 × 0.272 = 0.026		
	Sick people (0.459)	0.094 × 0.459 = 0.043		
	Medical evaluations (0.073)	0.094 × 0.073 = 0.007		
	Days of absence (0.141)	0.094 × 0.141 = 0.013		
	Mosquito breeding (0.175)	0.094 × 0.175 = 0.016		

^a^The final priorities of each alternative in terms of the chosen criteria. We multiply the value of the priority column from Table 2 by the value of the priority of the criteria in Table 1.

**Table 4:** The final ranks of the alternatives.*a*

Alternatives	Criteria					
	Cost	Time	Precision	Value	Refresh rate	Priority (AHP^b^)
Inspections	0.001	0.026	0.133	0.015	0.026	3 (0.201)
Sick people	0.019	0.003	0.030	0.255	0.043	1 (0.350)
Medical evaluations	0.009	0.013	0.084	0.126	0.007	2 (0.239)
Days of absence	0.009	0.008	0.022	0.044	0.013	5 (0.096)
Mosquito breeding	0.004	0.006	0.024	0.076	0.016	4 (0.126)

^a^The values for each alternative are repeated and the final priority of each one is calculated by the total sum for each row.

^b^AHP: analytic hierarchy process value.

**Figure 2:**
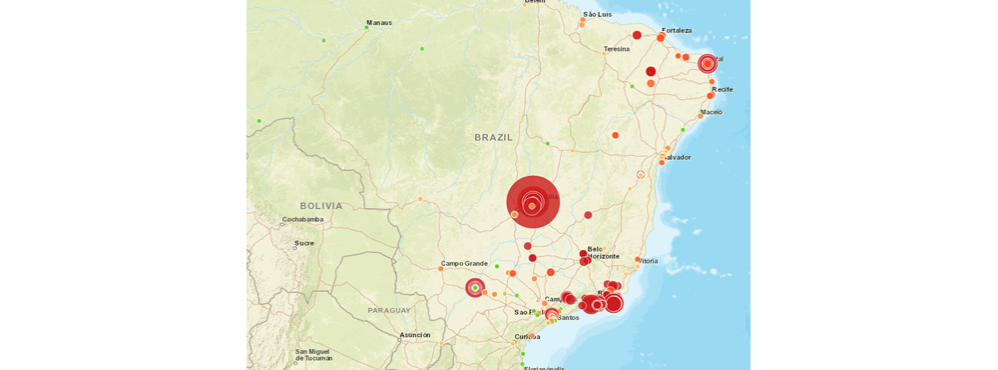
Risk map, considering the proposed index, for 2016, 2017, and 2018.

**Figure 3:**
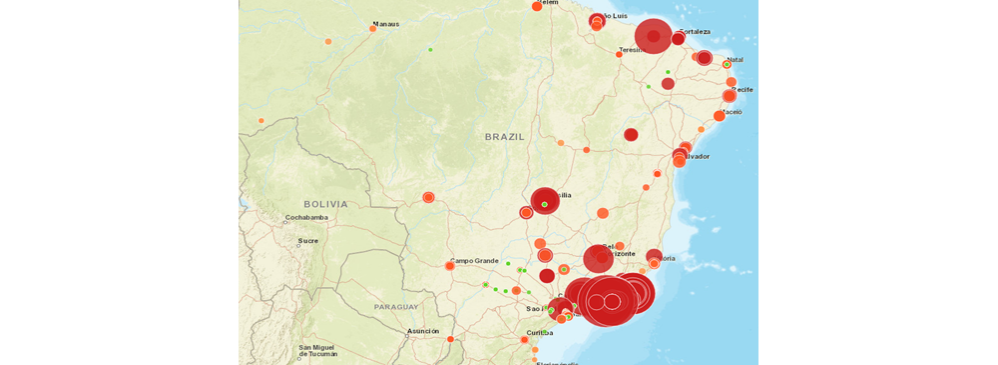
Risk map, considering only the number of mosquito breeding sites, for 2016, 2017, and 2018.

This new index is georeferenced by the inspection places retrieved from the data source, as seen in Figure 1. We used the results of inspections to indicate the future risk of mosquito breeding. The map of risks, considering the new index for 2016, 2017, and 2018, is presented in Figure 2. The visualization component lets specific regions be selected. The risk map considering only the number of mosquito breeding sites for the same period is shown in Figure 3. We note that the relevant regions in the graphs are strikingly different when comparing the maps. In both figures, the size of the bubble (the greater the risk, the larger the bubble) and its color (red indicates the highest risk) refer to the risk. The new index shows that the risk is not only concentrated in the southeast region (a richer region, where the number of inspections is higher) of Brazil but also is widely distributed across the country. However, the new index gives higher priority to the central region of Brazil, near Brasília. The extra data show that mosquito-breeding sites may be insufficient to provide a reliable index, and extra data may improve the risk prediction accuracy.

**Figure 4:**
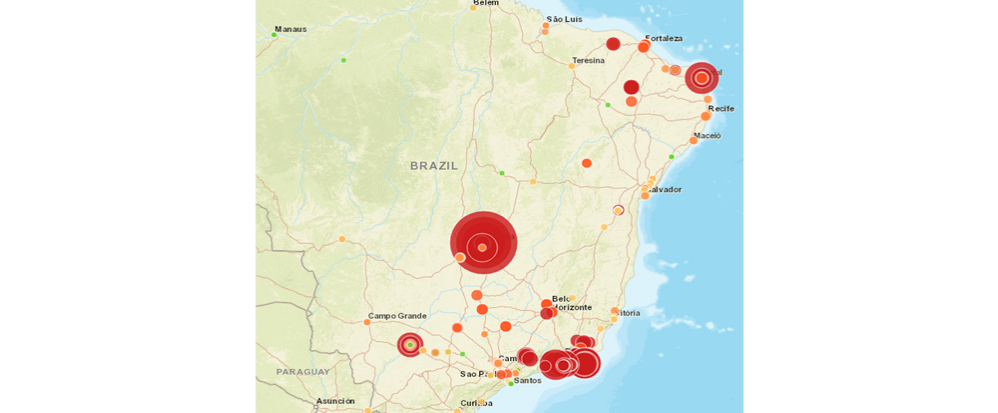
Risk map, considering the proposed index, for 2016 and 2017.

**Figure 5:**
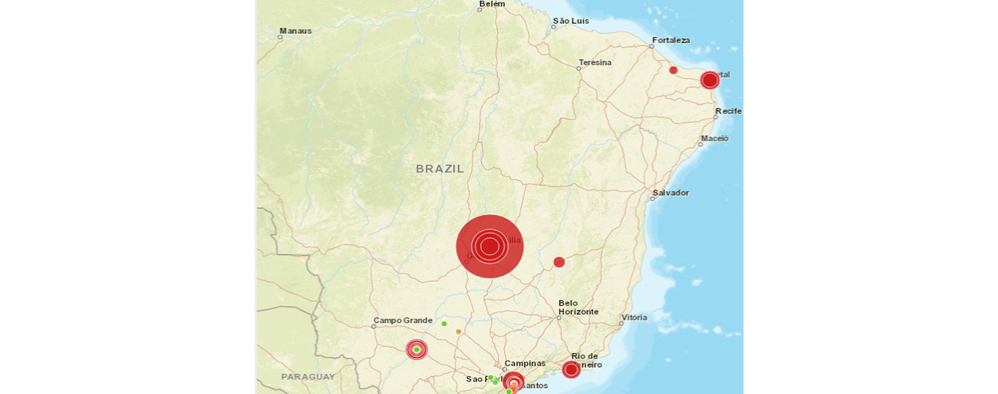
Number of sick people, considering only 2018.

Another interesting result is that a map elaborated with the new index can be more useful than the current map used by the decision makers (Figure 3) in predicting where the number of infections will be higher. This can be seen by analyzing Figure 4, which shows our proposed index considering data from 2016 and 2017, as well as Figure 5, which presents the number of cases in 2018. The regions where our index showed higher risks for 2016 and 2017 were mostly the same areas with the highest concentration of sick people in 2018.

## Discussion

### Contributions of This Study

In this work, we proposed a georeferenced index to help in the identification of likely risk areas for the proliferation of *A. aegypti* breeding sites in Brazil. We applied multi-criteria analysis, specifically AHP, to prioritize the metric alternatives based on 5 criteria. By using this methodology, we were able to produce risk maps for *A. aegypti* disease control. This is a well-stablished approach as shown by the related works [3,6-9,11-14]. We then compared the map currently produced for the Ministry of Planning, Development, and Administration of Brazil with the one created by our proposed index. The information shown by these maps was entirely different, given that we sought to indicate which areas have a higher risk of *A. aegypti* breeding sites, instead of the places with more inspections or breeding sites already identified. We also tested the index’s capacity for identifying areas affected by the diseases caused by *A. aegypti* and showed that it can help in identifying these areas. It is our understanding that the proposed index—with its future orientation—could be another tool to public health practitioners in preventing the appearance of new *A. aegypti* breeding sites and in showing how information technology can be applied to help solve public health challenges. Other initiatives such as participatory disease surveillance [29-31] can be used to provide additional data to be used as analysis source.

### Limitations

This work does not use environmental variables that affect the density and distribution of the mosquitoes (such as temperature, precipitation, air humidity, availability of water tanks), which can be added in further versions of this study. In this work, we were only able to georeference the index that resulted from the first AHP, which only involved the opinion of 3 of the authors. After presenting the results of this work as a poster at the Workshop on Big Social Data and Urban Computing that was held in the Very Large Databases Conference, we consulted a group of 3 experts on diseases caused by *A. aegypti*, regarding the use of AHP in conjunction with their opinions. A form was created to help fill out the AHP spreadsheet with the pairwise comparisons. The result of this effort, as well as a comparison between the executions of the AHP by the authors and experts, can be seen in Table 5. This new AHP with a group of experts on diseases caused by *A. aegypti* will allow us to improve our research.

**Table 5:** The final ranking of the alternatives by the experts and the authors.

Alternatives	Ranking by experts (AHP^a^)	Ranking by authors (AHP)	Difference
Inspections	3 (0.215)	3 (0.201)	0.014
Sick people	2 (0.254)	1 (0.350)	–0.096
Medical evaluations	5 (0.091)	2 (0.239)	–0.148
Days of absence	4 (0.123)	5 (0.096)	0.027
Mosquito breeding	1 (0.316)	4 (0.126)	0.19

^a^AHP: analytic hierarchy process value.

### Conclusions

The proposed approach uses unconventional indicators based on the presence of mosquitoes: number of inspections, number of sick people, number of medical evaluations, number of days of sickness absence, and number of mosquito breeding sites found. Thus, this work provides a novel paradigm for public health practitioners in preventing the appearance of new *A. aegypti* breeding sites. This new index may also be applied in conjunction with the current methodologies. Besides the criteria we used in our index proposal, we will explore environmental criteria that affect the density and distribution of the mosquitoes (such as temperature, precipitation, air humidity, availability of water tanks), which are the standard variables in the identification of *A. aegypti* breeding sites.

## Abbreviations

AHP: analytic hierarchy process
FMD: foot-and-mouth disease
GAIA: geometrical analysis for interactive aid
MAUP: modifiable areal unit problem
MCDA: multi-criteria decision analysis
PRADIN: program to support decision making based on indicators
PROMETHEE: Preference Ranking Organization Method for Enrichment of Evaluations
